# Supplementary material for: Splice-Junction-Based Mapping of Alternative Isoforms in the Human Proteome
Source: Cell Rep. Author manuscript; Available in PMC 2020 Jan 15. (PMC6961840; doi:10.1016/j.celrep.2019.11.026)
Supplement: 2 [file NIHMS1546469-supplement-2.zip › DF1/PXD010154/Testis/Testis_51_ADCY4_TGPPSATLPHLGLR.pdf]

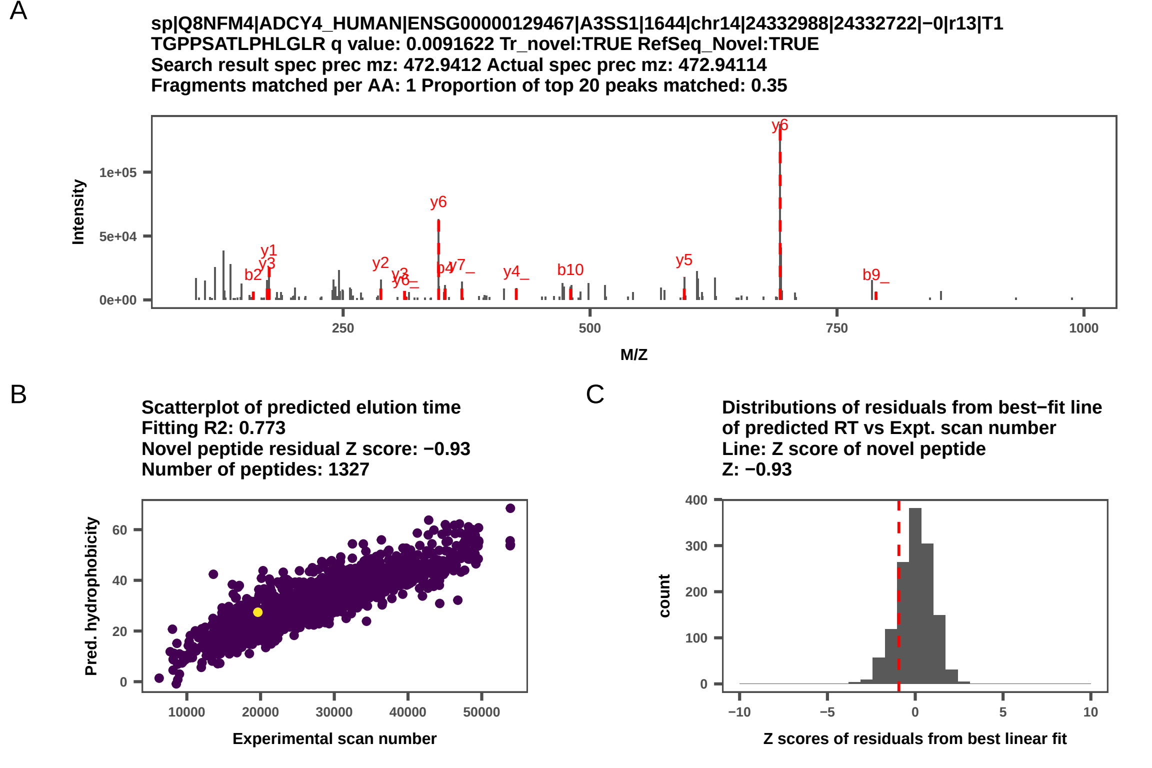
A
sp|Q8NFM4|ADCY4_HUMAN|ENSG00000129467|A3SS1|1644|chr14|24332988|24332722|−0|r13|T1
TGPPSATLPHLGLR q value: 0.0091622 Tr_novel:TRUE RefSeq_Novel:TRUE
Search result spec prec mz: 472.9412 Actual spec prec mz: 472.94114
Fragments matched per AA: 1 Proportion of top 20 peaks matched: 0.35
Intensity
1e+05
5e+04
0e+00
y6
y6
y1
y3
b2
y2
y3
y6_
b4
y7_
y4_
b10
y5
b9_
250
500
750
1000
M/Z
B
Scatterplot of predicted elution time
Fitting R2: 0.773
Novel peptide residual Z score: −0.93
Number of peptides: 1327
Pred. hydrophobicity
60
40
20
0
10000
20000
30000
40000
50000
Experimental scan number
C
Distributions of residuals from best−fit line
of predicted RT vs Expt. scan number
Line: Z score of novel peptide
Z: −0.93
count
400
300
200
100
0
−10
−5
0
5
10
Z scores of residuals from best linear fit
